# Rapid Induction of Liver Regeneration for Major Hepatectomy (REBIRTH): A Randomized Controlled Trial of Portal Vein Embolisation versus ALPPS Assisted with Radiofrequency

**DOI:** 10.3390/cancers11030302

**Published:** 2019-03-04

**Authors:** Long R. Jiao, Ana B. Fajardo Puerta, Tamara M.H. Gall, Mikael H. Sodergren, Adam E. Frampton, Tim Pencavel, Myura Nagendran, Nagy A. Habib, Ara Darzi, Madhava Pai, Rob Thomas, Paul Tait

**Affiliations:** 1 Department of Surgery & Cancer, Imperial College London, London W12 0HS, UK; a.fajardo-puerta@imperial.ac.uk (A.B.F.P.); tamara.gall1@nhs.net (T.M.H.G.); mikael.sodergren@nhs.net (M.H.S.); a.frampton@imperial.ac.uk (A.E.F.); t.pencavel1@nhs.net (T.P.); myura.nagendran@imperial.ac.uk (M.N.); nagy.habib@imperial.ac.uk (N.A.H.); a.darzi@imperial.ac.uk (A.D.); madhava.pai@imperial.nhs.uk (M.P.); 2 Department of Radiology, Imperial College London, London W12 0HS, UK; robert.thomas1@nhs.net (R.T.); paul.tait@imperial.nhs.uk (P.T.)

**Keywords:** ALPPS, RALPPS, ALPPS-RF, hepatic resection, portal vein embolisation, portal vein ligation

## Abstract

To avoid liver insufficiency following major hepatic resection, portal vein embolisation (PVE) is used to induce liver hypertrophy pre-operatively. Associating liver partition with portal vein ligation for staged hepatectomy assisted with radiofrequency (RALPPS) was introduced as an alternative method. A randomized controlled trial comparing PVE with RALPPS for the pre-operative manipulation of liver volume in patients with a future liver remnant volume (FLRV) ≤25% (or ≤35% if receiving preoperative chemotherapy) was conducted. The primary endpoint was increase in size of the FLRV. The secondary endpoints were length of time taken for the volume gain, morbidity, operation length and post-operative liver function. Between July 2015 and October 2017, 57 patients were randomised to RALPPS (n = 29) and PVE (n = 28). The mean percentage of increase in the FLRV was 80.7 ± 13.7% after a median 20 days following RALPPS compared to 18.4 ± 9.8% after 35 days (*p* < 0.001) following PVE. Twenty-four patients after RALPPS and 21 after PVE underwent stage-2 operation. Final resection was achieved in 92.3% and 66.6% patients in RALPPS and PVE, respectively (*p* = 0.007). There was no difference in morbidity, and one 30-day mortality after RALPPS (*p* = 0.991) was reported. RALPPS is more effective than PVE in increasing FLRV and the number of patients for surgical resection.

## 1. Introduction

Hepatic resection remains the gold standard treatment for patients with primary or secondary liver tumours providing them with the only chance for long-term survival. In resectable cases, a major hepatectomy is often necessary to achieve a clear resection margin (R0), a major determinant factor for long-term survival [1,2,3]. Liver failure remains the principal cause of post-operative death following major hepatic resection. This is inevitably due to an insufficient future liver remnant volume (FLRV) to support post-operative liver function, coupled with poor function from prolonged systemic chemotherapy. It is generally agreed that the FLRV must be at least or over 25–35% of the liver volume in order to overcome these problems. This leaves only 10–29% of patients suitable for extended and staged hepatic resection at presentation [4,5,6,7]. With the increased use of neoadjuvant chemotherapy to downstage tumours [8], liver function can be affected as a result of chemotherapy-associated liver injury (sinusoidal obstruction syndrome or chemotherapy-associated steatohepatitis), making patients more prone to post-operative liver failure.

Over the years, various methods have been used to induce hypertrophy of the FLRV pre-operatively with the aim of reducing post-operative complications and increasing the proportion of patients suitable for resection. Portal vein embolization (PVE) is the standard technique used and can result in an increase in FLRV of 11.9–39% [9,10]. In 2012, Schnitzbauer et al. [11] proposed the alternative method of “Associating Liver Partition and Portal vein ligation for Staged hepatectomy (ALPPS)”, a name coined by E. de Santibañes and P.A. Clavien [12]. A much greater increase in FLRV volume of 74% was seen in a much shorter period in comparison with PVE. However, the morbidity reported from this procedure is 33–58% [13,14,15], compared to 16% after PVE [9]. Since its introduction, ALPPS has become considered as a true surgical innovation of the 21st century [16]. On the other hand, due to increased morbidity and mortality [17], there are questions about its validity. Furthermore, the functional gain associated with the volume increase over a few days remains debatable. To capitalize the advantage of ALPPS without the additional risks, ALPPS assisted with radiofrequency (RALPPS) was described in addition to several other variant ALPPS [18,19,20,21,22]. The senior surgeon from our unit (L.R.J.) first described and introduced the concept of using radiofrequency (RF) to create a zone of ablation, which works as a “virtual” splitting for induction of liver hypertrophy as a variant ALPPS (RALPPS) [21], expanding upon the vast experience the author has had since the first publication of radiofrequency ablation (RFA) in management of liver tumours [23]. 

In 2015, a consensus meeting on ALPPS was held among international hepatic surgeons in Hamburg [24]. The conclusion was that further studies were needed before ALPPS should be used for routine pre-operative induction of FLRV for staged hepatic resections. Two randomised clinical trials (RCT) were proposed at this meeting: the multicenter LIGRO Trial from the Scandinavian group led by Sandström, comparing ALPPS and PVE [15]; and our own “regeneration of liver: portal vein embolization versus radiofrequency assisted ligation for liver hypertrophy (REBIRTH) trial”. The aim of our trial was to see if RALPPS could be safely performed to increase liver volume in a shorter period of time compared with the gold standard PVE. 

## 2. Materials and Methods

### 2.1. Study Design and Participants

This RCT was approved by the National Research Ethics Service (IRAS: 148741), and its protocol published and registered online (https://clinicaltrials.gov/ct2/show/NCT02216773). Patients with liver tumours requiring major resection were recruited after discussion at our liver multidisciplinary team (MDT) meeting in our tertiary referral centre for hepatopancreaticobiliary (HPB) surgery. All patients were routinely assessed by a triple phase, computed tomography (CT) scan of the chest, abdomen and pelvis, and a contrast-enhanced MRI of the liver. Patients with a FLRV to total liver volume (TLV) ≤25% and no preoperative chemotherapy and those with FLRV to TLV ≤35% and chemotherapy and/or prolonged course of chemotherapy corresponding to ≥10 cycles were considered for pre-operative manipulation of liver volume with either PVE or RALPPS. For patients with bilobar disease, a two-stage hepatectomy was considered as described in our unit [4]. All patients who had a WHO performance status of 0–2 without severe cardiopulmonary comorbidity were judged to be fit for major liver surgery. Those with extrahepatic metastases were excluded. Written informed consent was obtained from all patients before randomisation (Table 1). 

### 2.2. Randomisation

Randomisation was performed independently using http://www.sealedenvelope.com. There was no masking during the interventions, data collection or its analysis and interpretation. All patients underwent preoperative induction of liver hypertrophy with either RALPPS or PVE within two weeks of randomisation. 

### 2.3. Procedures

**RALPPS** was performed laparoscopically or robotically. Any patient with failed laparoscopic or robotic RALPPS, because of technical difficulty, was crossed over to the PVE group as per trial protocol to adhere to the gold standard procedure of PVE. The surgical technique for RALPPS has been described previously [18,21]. In brief, following ligation of the right portal vein, the demarcation between the left and right lobes of the liver was clearly visible. Next, radiofrequency ablation (RFA) splitting of the liver with either cool tip RFA (Covidien, Hampshire, UK) or laparoscopic H4X sealer (Laparoscopic Habib^TM^ 4X, Rita, CA, USA) was performed under the guidance of intraoperative ultrasound above the major intrahepatic pedicles, ranging from 40 mm to 60 mm in depth, for completion of RALPPS [25]. When an extended right hepatectomy was required for segment IV tumour(s), the RFA was undertaken in a similar manner but to the left of the tumour(s) on the right side of the falciform ligament if the tumour in segment IV was not resected at the stage 1. All patients had a restaging triple-phase CT scan requested two weeks after RALPPS to assess the FLRV prior to right or extended right hepatectomy.

**PVE** was performed by one of two senior radiologists (R.T., P.T.) as standard. Under local anaesthetic, percutaneous puncture of the ipsilateral portal vein was performed. Polyvinyl alcohol diluted in Iohexol (Omnipaque, GE Healthcare, Bucks, UK) was injected into each segmental branch of the right hemi-liver until stasis within the vessel was achieved. A combination of polyvinyl alcohol and coils was used to seal the track and to achieve PVE. To maximise the liver regeneration, additional segment IV branch was also embolised when necessary. PVE was performed during the same admission for stage-1 operations prior to discharge. Patients had a restaging triple-phase CT to assess volume changes six weeks later as per standard of the current practice. 

### 2.4. Hepatic Resection

Patients with >25 and >35% FLRV and had not or had preoperative chemotherapy, respectively, following RALPPS or PVE without extra-hepatic disease went on to have an open, laparoscopic or robotic, right or extended right hepatectomy, as previously reported [4]. In brief, for laparoscopic or robotic resection, a five-port technique was performed. For open hepatic resection, a standard right subcostal incision with an upper midline extension was used. A Harmonic scalpel was used for resection to ensure haemostasis during open or laparoscopic/robotic surgery (Ethicon Endo-Surgery, Cincinnati, OH, USA). For laparoscopic and robotic resection, the specimen was placed in a 15 mm Endocatch bag (Metronic, Watford, UK) and retrieved whenever possible via a Pfannenstiel incision. Standard placement of 20F Robinson drain at the resection margin was performed.

### 2.5. Measurement of Liver Volume

TLV, FLRV and total liver tumour volume (TLTV) were measured by a single author (A.B.F.P) and checked independently by a second author (T.M.G.). These volumes were assessed from the CT scans performed before and after RALPPS or PVE, and prior to hepatic resection. Liver volumes were calculated using ImageJ (Image Processing and Analysis in Java, National Institute of Health) as previously described [26]. The FLRV was defined as the proportion of FLRV to TLV minus TLTV (FLRV = FLRV/TLV − TLTV).

### 2.6. Outcomes

The primary endpoint was defined as the percentage increase in the FLRV. The secondary endpoints included 30-day morbidity and mortality and 90-day mortality, time to second operation, and liver function tests, haemoglobin and C-reactive protein (CRP) levels after completion hepatectomy on post-operative days 1, 3 and 5. Post-operative liver failure was defined as per the International Study Group of Liver Surgery: “the impaired ability of the liver to maintain its synthetic, excretory, and detoxifying functions, which are characterized by an increased international normalized ratio and concomitant hyperbilirubinemia on or after postoperative day 5” [27]. Surgical resection margins were compared between the two groups. A positive resection margin was defined as tumour within 1 mm of the resection margin.

### 2.7. Failure of Treatment

This was defined as any patient that failed to proceed to stage-2 hepatic resection for the following reasons.

1. Disease progression (local, regional or systemic).
2. Inadequate FLRV ≤25% for patients without preoperative chemotherapy, and ≤35% for those with preoperative chemotherapy.

### 2.8. Statistical Analysis

The sample size was based on the pilot data from our previous publication [21]. The sample size calculation assumed two-sided testing. The sample size of each arm was calculated using the equation designed for two proportions; α was set at 0.05 to control for type 1 error and β at 0.10 to control for type 2 error. Based on these data, a power calculation estimated a total sample size of 16 patients. However, because of the relatively small sample size of the pilot data, it was decided to aim to recruit 25 patients per group to the trial. Statistical analysis was performed using SPSS version 21 (IBM, Bristol, UK). Data are reported with intention to treat as the median and range or mean and standard deviation. Parametric data were compared using the student’s t-test, and non-parametric data with the Mann–Whitney U test for continuous data or Chi-square test for categorical data. 

## 3. Results

**Figure 1:**
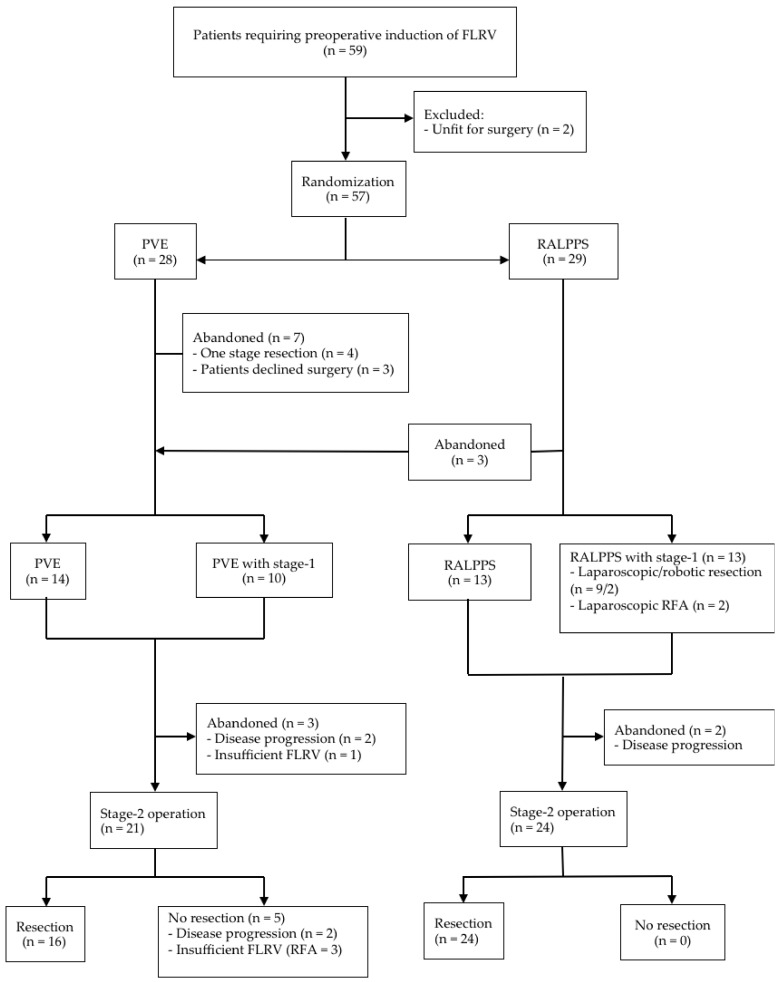
Trial profile. PVE: portal vein embolisation; RALLPS: liver partition with portal vein ligation for staged hepatectomy assisted with radiofrequency; RFA: radiofrequency ablation.

Between 1 July 2015 and 31 October 2017, a total of 59 patients were screened, and 57 underwent randomisation (Figure 1). In the PVE group, four cases were suitable for a one-stage operation after reassessment, and three patients declined to be involved in the trial. The median follow-up time was 24 months (3–33 months). No patient was lost during follow up. The characteristics of patients and tumours in each group were similar (Table 2). 

The majority of patients had colorectal liver metastasis (CRLM, 79.2% in PVE group vs. 76.9% in RALPPS group) with a median of two metastases for each patient in the PVE group, and three in the RALPPS group, with an average size of 45.2 ± 30.1 mm versus 52.2 ± 37.7 mm, respectively (*p* = 0.53). All CRLM patients had more than 6–8 cycles of standard systemic chemotherapy, some having <10 cycles and some having ≥10 cycles (n = 1 and n = 18 in PVE; n = 2 and n = 18 in RALPPS), respectively. 

Among 26 patients who underwent the first stage-1 operation in the RALPPS group, 13 had bilobar disease and underwent additional liver resection and RFA to tumours in the FLRV, consisting of tumourectomy in segment II (n = 3), segment III (n = 4) and segment IV (n = 4), respectively, and RFA to tumours (n = 2) (Figure 1). The median length of operation was 90 min. There was median intraoperative blood loss of 310 mL with no perioperative blood transfusion. Three patients in the RALPPS group had diagnostic laparoscopy only, which revealed extensive adhesions in hepatic hilum from previous laparotomy (n = 2) and a large tumour from segment VI and V obliterating the view and structures of hepatic hilum (n = 1). The procedure was abandoned, and all three patients were crossed over into the PVE group as per trial protocol to adhere to the standard treatment. The median total length of hospital stay (LOS) was three days. Minor complications (Dindo I to IIIa) occurred in 23.0% patients. No patients developed a post-operative bile leak. One patient with endometrial carcinoma developed acute compartment syndrome in her right lower limb 4 hours postoperatively as a result of vascular injury from a femoral puncture for vascular access on anaesthetic induction (Dindo IIIb).

PVE was performed in 24 patients, with 10 patients undergoing a stage-1 laparoscopic tumourectomy for bilobar disease from segment III (n = 7) and segment IV (n = 3), respectively. Minor complications (Dindo I to IIIa) occurred in 20.1% patients (Table 2). The length of hospital stay (LOS) in both groups was similar (Table 2, *p* = 0.06). To maximise the liver regeneration, an additional segment IV branch was also embolised (n = 18). RALPPS was able to significantly increase the FLRV in a much shorter length of time when compared to PVE (80.7 ± 13.7% vs. 18.4 ± 9.8%, *p* < 0.001 and 20.0 ± 5.6 days vs. 41.6 ± 15.5 days, *p* < 0.001, respectively) (Table 3). Following restaging CT scans, 21 patients in the PVE group and 24 in the RALPPS group underwent stage-2 liver resections. At laparotomy, five patients in the PVE group did not have resection because of disease progression, and the procedure was abandoned (n = 2), or because of insufficient increase in FLRV, and therefore only RFA was performed (n = 3). 

In the RALPPS group, all 24 cases underwent resection (Figure 1 and Table 2). When analyzed with intention to treat, there was a significant failure rate of PVE (PVE, 33.3% vs. RALPPS, 7.7%, *p* = 0.007), and fewer patients proceeded to the final hepatic resection after PVE (PVE, 66.7% vs. RALPPS, 92.3%, *p* = 0.007). The type of liver resection, operative time, amount of intraoperative bleeding, blood transfusion required, LOS and morbidity were not significantly different between the two groups (Table 2).

Furthermore, no significant difference in liver function blood tests was detected between the two groups on days 1, 3 and 5 post-hepatectomy (Supplementary Table S1). In the RALPPS group, serious complications (≥Dindo 3b) occurred in three patients, consisting of an intra-abdominal collection which was drained radiologically but also associated with renal failure needing haemofiltration (n = 1), supraventricular tachycardia and chest infection with pleural effusions requiring radiological drainage (n = 1), and a prolonged post-operative ileus successfully managed with total parenteral nutrition (n = 1). One patient died from peritonitis secondary to ischaemic gut 10 days after open right hepatectomy (3.8%) (Table 2). In this patient, at re-laparotomy on day 7 post-hepatic resection, a perforated loop of twisted ischaemic small bowel was found in the right hepatic space, most likely caused by the rotation of mesentery, as the small bowel reached upwards to fill the empty space following right hepatectomy. The patient had 20 cm of mid small bowel resected, with an end-to-end primary anastomosis, but died three days later from a sudden cardiorespiratory arrest. The resection margin positivity was not significantly different between the two groups (*p* = 0.71). In the PVE group, serious complications occurred in one patient who developed chest infection with pleural effusion eight days postoperatively, requiring intervention.

## 4. Discussion

This is the first randomized controlled trial comparing a new innovative method for rapid induction of liver hypertrophy (RALPPS) with gold standard PVE for pre-operative manipulation of FLRV prior to major hepatectomy, and the second only randomised controlled trial on ALPPS or any of its modifications since the procedure was first proposed in 2012. Our results showed that RALPPS produced a significantly greater increase in liver volume and within a much shorter time period compared to PVE, without increased morbidity and mortality.

To allow more patients with initially unresectable liver disease to successfully undergo hepatic resection, two established methods are currently used to induce hypertrophy of the FLRV: PVE and ALPPS or its variants. Both methods involve occlusion of the portal vein in the liver lobe to be resected to induce atrophy in this lobe with subsequent hypertrophy in the contralateral lobe by diverting the portal venous flow into the FLRV. A new third option for induction of liver hypertrophy, i.e., liver venous deprivation, has been described, reporting to achieve a volume increase comparable to ALPPS by simultaneous embolisation of both right portal and hepatic veins [28]. A meta-analysis from our group, including 1088 patients who underwent PVE prior to hepatic resection [9], showed a mean hypertrophy rate of the FLRV after PVE of 11.9% after an average of 29 days, while in other cases, a volume increase as high as 39% could be achieved [10]. Major morbidity from PVE was seen in 2.2%, with no mortality. After hepatic resection, the morbidity rate was 16% with a 1.7% mortality rate. 

In 2012, Schnitzbauer et al. proposed an alternative method to PVE: a right portal vein ligation combined with in situ liver splitting in small-for-size settings [11]. The mean hypertrophy rate of the FLRV was 74%. However, morbidity was seen in 68% of the patients, with a post-operative bile leak of 24%. The mortality rate reported was 10%. To avoid bile leak, the group described a technique consisting in wrapping the whole diseased ischaemic liver in a hermetic plastic bag, which was subsequently reported in a letter to editor [12,29]. However, the morbidity rate remained high at 58% [30]. Given the high morbidity reported with ALPPS, an international online registry (http://www.alpps.net) was set up. Data from this registry showed a liver failure rate (by ISGLS criteria) and a mortality rate of 30% and 8.8%, respectively [31]. In order to minimize the associated complications reported with this technique, various alternative methods have been proposed and named as variant ALPPS (tourniquet ALPPS, mini-ALPPS, partial ALPPS, hybrid ALPPS and our own RALPPS) [18,19,20,21,22,32,33]. Although there was no clear evidence that ALPPS or its variants should replace PVE or that variant ALPPS were superior to conventional ALPPS [24], there seemed to be a significant reduction in morbidity and mortality rates when comparing variant ALPPS with conventional ALPPS [18]. Our randomised controlled trial was designed to address some of these needs. 

In the current study, a high hypertrophy rate of 80.7 ± 13.7% in the RALPPS group compared to 18.4 ± 9.8% in the PVE group was seen, which is similar to that reported after ALPPS [9,10] and also consistent with Sandström et al. findings showing 68.0 ± 38.0% increase in FLRV post ALPPS (Table 4) [15]. We achieved this with a similar morbidity rate when compared with PVE. No bile leaks were seen in patients following stage-1 hepatic resection with RALPPS. The final stage-2 resection was achieved in all RALPPS patients, but failed in five PVE patients, in three of them because of inadequate hypertrophy of the FLRV. Within the PVE group in our study, 18 patients underwent an additional segment IV branch embolization in keeping with the report from the MD Anderson group where a significant improvement was observed in segments II and III hypertrophy compared with right PVE alone without increased complications [34].

A reduced time period between operations may lead to fewer adhesions and operative difficulty in cases where stage-1 operation is required to remove left-sided tumours prior to PVE and ALPPS or its variants. There is great debate with regards to the timing of hepatic resection following PVE and ALPPS or its variants, as there is currently no widely available technique able to predict the hepatic synthetic function of the FLR. Therefore, the volumetric increase in FLRV is used as a crude measurement for determining its future function preoperatively in non-cirrhotic liver. However, a rapid increase in liver volume within a short period of time represents liver hypertrophy as a result of hypertrophy of hepatocytes (a volume increase), as shown in animal studies, rather than hyperplasia (a true functional gain) [35]. The data from the ALPPS registry also seemed to report a high liver failure rate of 30% following hepatectomy [31]. As a result, most liver surgeons would still prefer staged liver resection with PVE to give more time to gain true liver hyperplasia following hypertrophy and therefore function, prior to major liver resection [36,37]. Our approach towards this is similar, with a median of 20 days prior to repeat CT scan and subsequent hepatic resection to allow time for liver regeneration and a true functional gain, rather than simply a volume increase. Although not clearly reflecting the liver regeneration process nor regional liver function, when biochemical liver function tests post-hepatectomy were compared, there was no difference compared to PVE. 

The physiological mechanism for this greater increase in FLRV in ALPPS is not fully understood, but it may be related to the response to the transection of the parenchyma, thereby reducing any shunting or collateralization [38,39]. In our case, a virtual splitting of liver parenchyma was achieved by creating a zone of necrosis along the intended future resection plane separating left from right lobe of liver. Furthermore, evidence suggests that RFA itself could greatly enhance liver regeneration in animal models, when compared with PVE alone [40]. Both these mechanisms may create a regeneration stimulus. The rapid regeneration response of a median of 20 days in our RALPPS group and 9 days in the original ALPPS paper, compared with 35 days in our PVE group has certain benefits. Importantly, there is less time for additional micro- and macro-metastatic disease to develop during the period of no-treatment whilst waiting for liver regeneration to occur. Indeed, in two patients, hepatic resection was not possible after PVE because of tumour progression.

The major drawback to ALPPS is the high morbidity and mortality rates, in particular from liver failure and bile leaks. ALPPS appears to have less function gained than expected by volume. These factors have prohibited the widespread clinical application of this surgical technique among some hepatic surgeons. We believe that RALPPS is a better alternative to ALPPS because it limits the invasiveness of the first stage, whilst capitalising on the liver hypertrophy, without high morbidity and mortality rates. In addition, RALPPS can be performed laparoscopically or robotically and, at the same time, as stage-1 hepatic resection in patients requiring a staged resection. However, in three cases, RALPPS were not attempted due to extensive hilar adhesions from previous operations (n = 2) and a large tumour obliterating the hilar view (n = 1). One might have then proceeded to an open approach to the hilus by performing RALPPS. However, our philosophy is that the stage-1 operation should be performed with less invasiveness to avoid surgical morbidity jeopardising the chance for a curative stage-2 hepatic resection. Hence, when the study was designed, we deliberately set out to perform a minimally invasive procedure to try to match the inherited advantage of a percutaneous PVE and adhere to the gold standard practice, without creating any added unfavourable circumstances inherent in an open surgical procedure. 

Although this trial showed a significant increase in FLRV within a shorter period of time following RALPPS when compared with the gold standard PVE, certain limitations have to be taken into consideration. Firstly, as the primary end point was volume change, different liver tumours types where included. Secondly, the allocation to the different arms was performed within two weeks of the HPB MDT meeting decision in order to arrange the procedures appropriately as per protocol. Thirdly, FLRV was used as a liver volumetric measurement instead of other available methods such as the standardized FLR using the Vauthey formula, the ratio of FLRV to body weight or the kinetic growth ratio [41,42,43]. Furthermore, no preoperative functional evaluation with mebrofenin scintigraphy or indocyanine green test was performed in our group of non-cirrhotic patients. Hence, as volume does not equal function and liver volumetric variations may not be a true indicator of liver synthetic function, this may be a weakness of the study. For PVE, other materials such as glue have shown to achieve a higher volume increase due to a better portal vein occlusion and lesser tendency to re-vascularization [10,44,45]. Another limitation is that there was no cross-overs from PVE to RALPPS within the PVE group. A drop-out of three patients from RALPPS over to the PVE group could have been avoided by converting the laparoscopic approach to an open operation, which might also result in a statistical limit of this study. As mentioned, no patients from the PVE group were crossed over to the RALPPS group, as the trial was designed deliberately to adhere to the standard clinical practice of using PVE for preoperative induction of liver hypertrophy. In light of the currently available level-1 evidence from this study and that of another group [15], we feel that patients whose FLRV failed to increase sufficiently for surgery following PVE should be considered to undergo RALPPS or ALPPS as a salvage procedure for induction of liver hypertrophy, to increase the number of these patients for curative liver resection.

## 5. Conclusions

This is the second only randomized controlled trial on ALPPS or any of its modifications since the procedure was first proposed in 2012. RALPPS can be performed at the same time as stage-1 hepatic resection, either laparoscopically or robotically to minimise surgical trauma to patients prior to stage-2 hepatic resection with comparable morbidity and mortality associated with PVE but a much greater increase in FLRV over a much shorter period of time.

## Figures and Tables

**Table 1:** Selection criteria.

**Inclusion Criteria**	Age ≥ 18 years Any patient requiring right or extended right hepatectomy with preoperative FLRV/TLV ≤25% in patients without preoperative chemotherapy ≤35% in patients with preoperative chemotherapy WHO performance status 0, 1 or 2 Patient able to comply with protocol requirements and deemed fit for surgical resection Written informed consent
**Exclusion Criteria**	Inability to give informed consent Pregnancy WHO performance status 3 or 4 New York Heart Association Classification Grade III or IV

FLRV: future liver remnant volume; TLV: total liver volume.

**Table 2:** Demographic characteristics and outcomes of intervention.

	PVE	RALPPS	*p* Value
	(n = 24)	(n = 26)	
**Patient and Tumour Characteristics**			
Age (mean ± SD, in years)	64.3 ± 8.9	62.4 ± 10.2	0.49
Male (%)	12 (50.0)	15 (57.7)	0.78
Type of tumor (%)			0.06
CRLM	19 (79.2)	20 (76.9)	
ICC	4 (16.7)	0 (0)	
HCC	0 (0)	1 (3.8)	
Others *	1 (4.2)	5 (19.2)	
Bilobar liver disease (%)	9 (37.5)	13 (50.0)	0.06
Synchronous metastases (%)	11 (45.8)	9 (34.6)	0.57
Number of metastases (median, range)	2 (0–11)	3 (1–10)	0.18
Size of largest metastasis (median, range in mm)	43 (15–108)	39 (12–150)	0.53
Primary tumour in situ (%)	1 (4.2)	3 (11.5)	0.61
**Neoadjuvant Chemotherapy Data**			
Neoadjuvant chemotherapy (%) **	20 (83.3)	22 (84.6)	0.99
FOLFOX	7	7	
FOLFIRI	5	3	
FOLFOX + Ab	1	2	
FOLFIRI + Ab	4	6	
FOLFIRI + aflibercept	1	1	
Capecitabine	1	0	
Oxaliplatin with capecitabine	1	1	
POMB-ACE	0	1	
Paclitaxel + cisplatin	0	1	
Number of cycles			
<10	1	2	0.97
≥10	18	18	0.99
**Details of PVE and RALPPS**			
PVE/RALPPS without stage 1	14 (58.3)	13 (50.0)	0.89
PVE/RALPPS with stage 1			
Tumorectomy (lap †/robotic)	9/1	9/2	
RFA (lap/robotic)	0/0	2/0	
Length of operation (median, range in mins)	90 (60–180)	115 (60–225)	0.88
Blood loss (median, range in mls)	300 (10–450)	310 (20–480)	0.88
Perioperative blood transfusion ^&^ (%)	0	1 (3.8)	0.33
Post procedural complications (%)	5 (20.1)	6 (23.0)	0.20
Dindo 1	3	3	
Dindo 2	2	2	
Dindo 3b	0	1	
Length of stay (median, range in days)	2 (1–13)	3 (2–17)	0.06
**Details of RALPPS (n = 29, %)**			
Laparoscopic	n/a	24 (82.8)	
Robotic	n/a	2 (6.9)	
Abandoned	n/a	3 (10.3)	
**Details of Stage-2 Operation**			
Type of operation			
Right hepatectomy (open/lap/robotic)	8 (7/1/0)	18 (14/3/1)	
Extended right hepatectomy (open/lap/robotic)	5 (5/0/0)	5 (4/1/0)	
Right hepatectomy with wedge resection/RFA (open/lap/robotic)	3 (3/0/0)	1 (0/1/0)	
RFA	3	0	
Abandoned intraoperatively	2	0	
Length of operation (median, range in mins)	180 (100–420)	180 (110–390)	0.87
Blood loss (median, range in mls)	500 (50–2850)	300 (50–3200)	0.30
Perioperative blood transfusion ^&^ (%)	6 (25.0)	10 (38.5)	0.18
Postoperative complications (%)	14 (66.7)	14 (53.8)	0.75
Dindo 1	4 (19.0)	0	
Dindo 2	9 (42.9)	9 (34.6)	
Dindo 3a	0	1 (3.8)	
Dindo 3b	1 (4.8)	0	
Dindo 4a	0	2 (7.7)	
Dindo 4b	0	1 (3.8)	
Dindo 5	0	1 (3.8)	
90 day mortality (%)	0 (0)	1 (3.8)	0.99
Length of stay (median, range in days)	7 (5–27)	8 (4–32)	0.25
**Resection Margin (%)**			
R0	11 (68.7)	18 (75.0)	0.87
R1	5 (31.2)	6 (25.0)	0.71
R2	0	0	

* Others: PVE: duodenal adenocarcinoma (n = 1); RALPPS: pancreatic NET (n = 1), germ cell ovarian tumor (n = 1), endometrial carcinoma (n = 1), breast cancer (n = 1) and leiomyosarcoma (n = 1). † Laparoscopic. ^&^ Number of patients transfused. Keys: CRLM: colorectal liver metastases; HCC: hepatocellular carcinoma; ICC: intrahepatic cholangiocarcinoma. ** FOLFOX: folinic acid, 5-FU, oxaliplatin. FOLFIRI: folinic acid, 5-FU, irinotecan. Ab: bevacizumab or cetuximab. POMB-ACE: cisplatin, oncovin, methotrexate, bleomycin, actinomycin, cyclophosphamide, etoposide.

**Table 3:** FLRV before and after PVE or RALPPS.

	PVE	RALPPS	*p* Value
	(n = 24)	(n = 26)	
	(no chemo (4); chemo (20))	(no chemo (4); chemo (n = 22))	
**Time from First-Stage Operation to Second CT**	35 (21–75)	20 (14–36)	<0.001
(Median in days and range)			
**Pre-Intervention FLRV**			
(Mean ± SD)			
no chemo	23.7 ± 1.1	23.1 ± 1.2	0.74
chemo	33.1 ± 1.5	33.8 ± 1.1	0.2
**Post-Intervention FLRV**			
(Mean ± SD)			
no chemo	28.5 ± 9.4	44.6 ± 5.6	0.04
chemo	40.4 ± 6.6	59.4 ± 4.3	<0.001
**Increase FLRV Post-Intervention** (%)	18.4 ± 9.8	80.7 ± 13.7	<0.001

The FLRV was calculated depending on the type of hepatic resection needed to achieve tumor clearance by the proportion of future liver volume to TLV minus total liver tumor volume (FLRV = FLRV/TLV − TLTV). Chemo: preoperative chemotherapy; no chemo: no preoperative chemotherapy.

**Table 4:** Comparison of two randomised control trials: RALPPS versus ALPPS [15].

	RALPPS	ALPPS ^15^
	(n = 26)	(n = 48)
**Stage 1**		
Type of operation		
Open	0	48
Laparoscopic/Robotic	24/2	0/0
Length of operation (median, range, mins)	115 (60–225)	NA
Length of stay (median, range, days)	3 (2–17)	NA
Morbidity	23.0	NA
Mortality	0	NA
**FLRV Increase** (Mean ± SD, %)	80.7 ± 13.7	68.0 ± 38.0
**Time from Stage 1 to Stage 2** (Mean ± SD, days)	20.0 ± 5.6	11.0 ± 11.0
**Stage 2**		
Complications grade ≥ 3b (%)	15.3	11.0
30 (90) day mortality (%)	3.8 (0)	9.1(0)
**Total length of stay** (Mean ± SD, days)	15.3 ± 9.7	23.0 ± 17.0
**Resection Rates** (%)	92.3	92.0

NA: not available.

## Supplementary Materials

The following are available online at https://www.mdpi.com/2072-6694/11/3/302/s1, Table S1: Liver function, haemoglobin and CRP levels following major hepatic resection.
