# Supplementary material for: Rapid Induction of Liver Regeneration for Major Hepatectomy (REBIRTH): A Randomized Controlled Trial of Portal Vein Embolisation versus ALPPS Assisted with Radiofrequency
Source: Cancers (Basel). 2019 Mar 4;11(3):302. doi: 10.3390/cancers11030302 (PMC6468856; doi:10.3390/cancers11030302)
Supplement: Supplementary file 1 [file cancers-11-00302-s001.pdf]

cancers

MDPI

Article

Rapid induction of liver regeneration for major hepatectomy (REBIRTH): A randomized controlled trial of PVE vs. ALPPS assisted with radiofrequency.

Long R. Jiao, Ana B Fajardo Puerta, Tamara MH Gall, Mikael H Sodergren, Adam E Frampton, Tim Pencavel, Myura Nagendran, Nagy A Habib, Ara Darzi, Madhava Pai, Rob Thomas and Paul Tait

Supplementary Material

Table S1. Liver function, haemoglobin and CRP levels following major hepatic resection.

	PVE (n = 16)	ALPPS-RF (n = 24)	*p* Value
	Bilirubin		
Pre-op	13.7 ± 14.2	9.9 ± 5.1	0.211
Post-op			
D1	34.9 ± 20.8	35.1 ± 25.8	0.982
D3	29.6 ± 10.6	36.9 ± 30.1	0.279
D5	34.0 ± 22.6	34.45 ± 29·3	0.961
	ALP		
Pre-op	151.2 ± 97.1	132.9 ± 89.7	0.502
Post-op			
D1	98.5 ± 46.7	101.6 ± 52.8	0.849
D3	117.5 ± 56.7	115.7 ± 53.7	0.918
D5	229.8 ± 123.3	228.4 ± 151.1	0.976
	ALT		
Pre-op	50.7 ± 60.9	34.6 ± 22.7	0.243
Post-op			
D1	628.6 ± 292.1	532.3 ± 351.4	0.381
D3	458.4 ± 249.6	383.2 ± 268.4	0.388
D5	207.6 ± 108.6	162.6 ± 94.9	0.198
	Albumin		
Pre-op	35.9 ± 2.8	33.7 ± 5.4	0.132
Post-op			
D1	25.7 ± 6.7	27.6 ± 6.4	0.363
D3	29.6 ± 5.7	31.1 ± 7.1	0.477
D5	30.5 ± 5.9	31.7 ± 7.7	0.612
	Hb		
Pre-op	122.5 ± 28.3	127.3 ± 14.6	0.456
Post-op			
D1	105.9 ± 20.7	99.1 ± 22.9	0.362
D3	100.5 ± 20.8	95.9 ± 13.1	0.414
D5	102.5 ± 16.3	90.7 ± 21.2	0.077
	CRP		
Pre-op	37.2 ± 55.1	18.7 ± 53.7	0.283
Post-op			
D1	66.4 ± 35.8	55.6 ± 29.3	0.309
D3	139.8 ± 59.8	159.1 ± 69.9	0.380
D5	127.2 ± 61.2	116.6 ± 57.4	0.593

	PT		
Pre-op	10.7 ± 0.6	15.9 ± 10.7	0.385
Post-op			
D1	14.0 ± 2.2	13.5 ± 1.7	0.479
D3	13.8 ± 2.7	13.9 ± 2.0	0.847
D5	12.7 ± 2.5	12.7 ± 1.4	0.816
	APTT		
Pre-op	25.1 ± 2.7	28.1 ± 6.8	0.445
Post-op			
D1	26.4 ± 2.6	26.9 ± 2.9	0.597
D3	29.9 ± 4.4	29.3 ± 4.1	0.659
D5	27.6 ± 3.0	28.8 ± 3.7	0.386
